# Identification of an Additional Minor Pilin Essential for Piliation in the Archaeon *Methanococcus maripaludis*

**DOI:** 10.1371/journal.pone.0083961

**Published:** 2013-12-30

**Authors:** Divya B. Nair, Daniel K. C. Chung, James Schneider, Kaoru Uchida, Shin-Ichi Aizawa, Ken F. Jarrell

**Affiliations:** 1 Department of Biomedical and Molecular Sciences, Queen’s University, Kingston, Ontario, Canada; 2 Department of Life Sciences, Prefectural University of Hiroshima, 562 Nanatsuka, Shobara, Hiroshima, Japan; Université Claude Bernard - Lyon 1, France

## Abstract

*Methanococcus maripaludis* is an archaeon with two studied surface appendages, archaella and type IV-like pili. Previously, the major structural pilin was identified as MMP1685 and three additional proteins were designated as minor pilins (EpdA, EpdB and EpdC). All of the proteins are likely processed by the pilin-specific prepilin peptidase EppA. Six other genes were identified earlier as likely encoding pilin proteins processed also by EppA. In this study, each of the six genes (*mmp0528, mmp0600, mmp0601, mmp0709, mmp0903* and *mmp1283*) was deleted and the mutants examined by electron microscopy to determine their essentiality for pili formation. While mRNA transcripts of all genes were detected by RT-PCR, only the deletion of *mmp1283* led to nonpiliated cells. This strain could be complemented back to a piliated state by supplying a wildtype copy of the *mmp1283* gene in trans. This study adds to the complexity of the type IV pili system in *M. maripaludis* and raises questions about the functions of the remaining five pilin-like genes and whether *M. maripaludis* under other growth conditions may be able to assemble additional pili-like structures.

## Introduction

Type IV pili are a very common type of surface appendage found in a variety of Gram-negative and Gram positive bacteria, as well as certain members of the Domain Archaea [1]–[6]. They are involved in a wide variety of processes including adherence, aggregation, DNA transfer in transformation and conjugation, biofilm formation, electron transfer and a type of surface motility termed twitching [5]–[8]. The core components of a type IV pili system includes structural proteins with class 3 signal peptides, a prepilin signal peptidase, one or more ATPases and a conserved membrane (platform) protein [5], [9], [10]. One ATPase powers the incorporation of new subunits into the growing filament while in many cases, the presence of a second, depolymerizing ATPase acts to remove subunits from the structure. The combined activities of the two ATPases results in extension and retraction of the pili, leading to the twitching motility associated with type IV pili in many bacteria [11]. The conserved inner membrane or platform protein is considered to interact with the ATPase(s) and form an export complex for the structural proteins and to be involved in both pilus assembly and disassembly [10]. In addition to these conserved components, type IV pili systems in different organisms often have other components whose role in pilus assembly and function remain unknown [6].

The structural subunits of the type IV pilus consist of a major pilin and typically several other pilins, termed minor pilins due to their much lower abundance, all synthesized initially with class 3 signal peptides that are specifically processed by the prepilin peptidase [12], [13]. Minor pilins have been shown to be necessary for pili formation in several different systems [14]–[17] and they have been detected in sheared pili samples of *N. gonorrhoeae* and *P. aeruginosa* [16], [18], although other roles for minor pilins as activators of pilus assembly without incorporation into the structure have also been proposed in other systems [19], [20]. In some type IV pili systems, evidence for a minor pilus constituent acting as a specific adhesin has been presented [21]–[24].

Archaea are known to use the type IV pili-like model to assemble numerous surface structures [2], [3], [25], [26] including type IV-like pili [25], [26], [29]–[32], the bindosome for substrate uptake in *Sulfolobus solfataricus* [25], [33], [34]),likely the unusual Iho670 fibres of *Ignicoccus hospitalis* [35], [36] and the best studied example, namely the archaellum [25], [28], [37]–[40]. The name “archaellum” has been proposed to replace the term “archaeal flagellum” [27] since the archaeal structure, while involved in swimming (as well as other functions), is not homologous to the bacterial flagellum and is related instead to type IV pili in structure and likely assembly [28], [41], [42]. This proposal is still under discussion in the scientific community and further arguments, both pro and con, have been presented [43], [44].

Recent studies in *Methanococcus, Haloferax* and especially *Sulfolobus* have been devoted specifically to the study of the type IV-like pili [29]–[31], [45]–[48]. *Sulfolobus* has been shown to produce two different type IV pili structures. One, called UV-inducible type IV pili (Ups pili; [29], [47]), is widespread throughout the *Sulfolobales* while the second called archaeal adhesive pili (Aap pili) is limited so far to *S. acidocaldarius* [25], [30], [49]. Ups pili are upregulated under conditions that lead to DNA double stranded breaks such as UV light and their formation leads to cell aggregation that promotes DNA exchange that might help in repairing the DNA damage [29], [47]. On the other hand, Aap pili are the most abundant surface appendage observed on *S. acidocaldarius* under normal growth conditions in nutrient rich medium [25]. Aap pili are adhesion structures primarily but they also influence biofilms, promoting the formation of tower-like structures [49]. The loci identified as encoding the biosynthesis of both Aap pili and Ups pili were shown to consist of only five genes [2], [29], [30]. In each case, there are genes for two prepilins, a single pilin assembly ATPase, the conserved pilus membrane/platform protein and one additional gene in each operon that has an unknown function. In the Aap system, AapB appears to be the major pilin and AapA the minor pilin [30]. Mutational studies demonstrated that all five *aap* genes were necessary for pili formation [30]. In the Ups system, deletion of the gene encoding the ATPase (*upsE*) eliminated UV-induced aggregation [29]. The assignment of UpsA or UpsB proteins as the major or minor pilins has not been reported.

In *Haloferax volcanii*, six novel type IV pilins termed PilA(1–6) involved in adhesion have been recently studied [48]. Each protein contains a highly conserved domain of unknown function (Duf1628) at their N-terminus and all are processed by PibD. Strains carrying deletions of up to five of the six pilin genes are still able to adhere while a mutant with all six genes deleted cannot. Complementation of the strain deleted for all six pilin genes with any single pilin gene results in cells that have functional pili.

In *Methanococcus maripaludis*, an in-silico study by Szabo et al [32] identified the existence of a type IV pili-like locus, consisting of 11 potential genes, including three encoding prepilin-like proteins and one encoding a prepilin peptidase. Subsequent genetic studies demonstrated that two of the pilin genes (*epdB* and *epdC*) were essential for pili formation while deletion of the third (*epdA*) resulted in cells with a reduced number of pili [31]. Unexpectedly, none of these genes encoded the major structural protein which was revealed by mass spectrometry of purified pili samples to be MMP1685, located at a distant locus [31]. Several other genes in the 11 gene operon are also essential for piliation (Nair et al. submitted). Unlike the case with *Sulfolobus* pili loci, the assembly ATPase and conserved pilus membrane protein genes are not found within this operon (Nair et al., submitted). The novel additional essential genes found in the pilus locus in *M. maripaludis*, the presence of at least four pilin structural proteins, coupled with the separate locations of both the major pilin and the ATPase and membrane component genes and the presence of two essential copies of the membrane component gene (Nair et al., submitted) all suggest that the pili of this methanogen may be more complex than those found in *Sulfolobus* species.

In addition to the four identified pilin genes (*epdA*, *epdB, epdC* and *mmp1685*), the initial in-silico study also identified six other genes in the *M. maripaludis* genome that were predicted to be type IV pilin-like genes processed by EppA (*mmp0528, mmp0600, mmp0601, mmp0709, mmp0903 and mmp1283*) [32]. Whether these genes encode additional essential structural components of the type IV pili assembled mainly from MMP1685 subunits has not yet been addressed. These genes were the focus of the current study where it was shown that only *mmp1283* was essential for pili formation.

## Materials and Methods

### Strains and Growth Conditions

*Methanococcus maripaludis* MM900 [50]) and a non-archaellated Δ*flaK* mutant strain derived from MM900 [51] were grown in Balch medium III [52] at 35°C under a headspace gas of CO_2_/H_2_ (20∶80). For transformations, cells were grown in McCas medium [50] supplemented at various steps with neomycin (1 mg/ml) or 8-azohypoxanthine (240 µg/ml) for selection. For complementation experiments, transformants were grown in Balch medium III with added puromycin (2.5 µg/ml) to select for uptake of the vectors. *E. coli* TOP10 cells (Invitrogen), used for various cloning steps, were grown in Luria-Bertani medium supplemented with ampicillin (100 µg/ml) as needed for transformations.

### Reverse Transcriptase-PCR

RT-PCR was done to determine if all the six genes used in this study were being transcribed. Primer pairs were designed to amplify an internal fragment of each particular gene. The template RNA was extracted from the wildtype cells using an RNeasy Mini Kit (Qiagen Inc. Canada Mississauga, ON) with optional DNase digestion as per the manufacturer’s instructions. A One-Step RT-PCR kit (Qiagen Inc.) was used to amplify the cDNA. Using the same primer pairs, additional templates were also used in PCR reactions, including purified RNA not subjected to the reverse transcriptase step (as a control for DNA contamination of the RNA preparation) and genomic DNA to verify amplicon size and specificity of the primers.

### Construction of Gene Deletion Plasmids

Plasmids were generated as described earlier [50], [53] to make inframe deletions of each of the targeted potential minor pilin genes. The P1/P2 primers (Table 1) were used to amplify an approximately 1 kb fragment upstream of the target gene and the P3/P4 primers to amplify an approximately 1 kb fragment downstream of the gene. P2 and P3 primers were designed with added *Asc*I restriction sites so that the upstream and downstream fragments could be digested with *Asc*I and then ligated together, resulting in an inframe deletion of most of the targeted gene. The ligated piece was used as template for another PCR using the P1 and P4 primers, which were designed with added *Bam*HI sites. The approximately 2 kb PCR product was purified, digested with *Bam*HI and cloned into pCRPrtNeo to create the plasmids (Table 2) used in generating the deletion strains [50].

**Table 1:** Primers used in this study.

Technique and primer name	Sequence	Restriction sites
**RT-PCR**		
mmp0528_ RT_ for	5′-AATGGAAATGGGAATCTTGGTTG	
mmp0528_ RT_ rev	5′-AACGTTTATAAGGGCATTACTCG	
mmp0600_ RT_ for	5′-GAGACATATTATCCGACGTTAGG	
mmp0600_ RT_ rev	5′-GTGATGTTTCAGTATATGGTAACAG	
mmp0601_ RT_ for	5′-GGAACGATATTCGCGGCACACATG	
mmp0601_ RT_ rev	5′-CTGGGTGGAACATCATAGGTAAGG	
mmp0709_ RT_ for	5′-GAGACTACTGCTGTTAATGATGTTCAGG	
mmp0709_ RT_ rev	5′-CGTAAGTAGTACTGTCCAGCACCAGTTC	
mmp0903_ RT_ for	5′-CCAGATATCTCTTGAACTTGGTG	
mmp0903_ RT_ rev	5′-CTGCACTGTTATACATGCCGACAG	
mmp1283_ RT_ for	5′-GCTGGTTCTTGCGGTCATTACAG	
mmp1283_ RT_ rev	5′-AAGCATCTATTGCTGTATTGTGAG	
**Screening of deletion mutants**		
mmp0528_ seq _ for	5′-GGATCATGGGGAGATGACCC	
mmp0528_ seq _ rev	5′-GTGCACGGCATACATTCGTG	
mmp0600_ seq _ rev	5′-TACTGGCGACTATTATACATTGG	
mmp0600_ seq _ rev	5′-GAAATAGTATTTGCGTCACTAGTTCCG	
mmp0601_ seq _ for	5′-TGACTACGTGATTATTGGAAGAG	
mmp0601_ seq _ rev	5′-GTATTAATTCTATCGAAATCTGG	
mmp0709_ seq _ for	5′-GAGTATGCCCTCATGGGGTAGC	
mmp0709_ seq _ rev	5′-GTTCCGTCACGGTAATTACC	
mmp0903_ seq _ for	5′-CCGAACCTAAATTTAGGAGG	
mmp0903_ seq _ rev	5′-CCGAGAATTGCACCTGTTGCG	
mmp1283_ seq _ for	5′-GAAGGTTGTATTGGGTTTAC	
mmp1283_ seq _ rev	5′-GAGACATGGTACTGCAATCG	
**In-frame deletions/plasmids**		
mmp0528_P1	5′-CGGATCCGGATATTACCGAAAGGAAAGT	BamH1
mmp0528_P2	5′-TTGGCGCGCCAATGTTTGTGGCAATATTTCTG	Asc1
mmp0528_P3	5′-TTGGCGCGCCCAACCAAGATTCCCATTTCC	Asc1
mmp0528_P4	5′-GCGGATCCACATTTCTGCTCACAATCTTCG	BamH1
mmp0600_P1	5′-GCGGATCCGGCACACATGACAAAGAATACTGTAAGC	BamH1
mmp0600_P2	5′-TTGGCGCGCCTACCTGCAAGAAGTACTCTGTC	Asc1
mmp0600_P3	5′-TTGGCGCGCCACAACTTCGGGGACTATAGAGCTG	Asc1
mmp0600-P4	5′-GCGGATCCTGCTGGATTATATTCCTGATGATGAGG	BamH1
mmp0601_P1	5′-GCGGATCCTTCTCGCGGAGTCCTAACTCTTGTTGG	BamH1
mmp0601_P2	5′-TTGGCGCGCCTCATGTGTGCCGCGAATATCG	Asc1
mmp0601_P3	5′-TTGGCGCGCCACTGGCGACTATTATACATTGG	Asc1
mmp0601_P4	5′-GCGGATCCTTCCACCAAAGTCTGTTGTGGTGGTT	BamH1
mmp0709_P1	5′-GCGGACTGCGGATCCGTGGACTCGG	BamH1
mmp0709_P2	5′-TTGGCGCGCCCTAGTGCAACGTAAGAACCAG	Asc1
mmp0709_P3	5′-TTGGCGCGCCCAGTACTACTTACGATTAGTGG	Asc1
mmp0709_P4	5′-GCGGATCCCGCAACTGCAATTCCAAGTCCG	BamH1
mmp0903_P1	5′-GCGGATCCAATCCGTGGCCTTTAATTGGTGG	BamH1
mmp0903_P2	5′-TTGGCGCGCCGTTCAAGAGATATCTGGCCCCG	Asc1
mmp0903_P3	5′- GTGGCGCGCCAGTGCAGTAAACAGAATTACTG	Asc1
mmp0903_P4	5′-GCGGATCCAATTCGTCGCTTCCAACCATG	BamH1
mmp1283_P1	5′-GGGGATCCAGTCTTCCGGTTCCAACTCTTAACG	BamH1
mmp1283_P2	5′-TTGGCGCGCCAAATTCAAGACTCAATTGGC	Asc1
mmp1283_P3	5′-TTGGCGCGCCTGCAATAGATGCTTTAAGTGAAGTTAG	Asc1
mmp1283_P4	5′-GGGGATCCGGGCGGAATTCCCGTTGAGGAC	BamH1
**Complementation**		
mmp1283_comp_for	5′-CCAATGCATGTCTGTTGCTTTAAAGAAGTTTTTTTCGAA ACG	Nsi1
mmp1283_comp_rev	5′-AGCACGCGTTTAACTAACTTCACTTAAAGCATCTATTGC	Mlu1

**Table 2:** Plasmids used in this study.

Plasmid	Description and/or genotype	Source or reference
pCRPrtNeo	*hmv* promoter-*hpt* fusion plus Neo^r^ cassette in pCR2.1Topo; Amp^r^	[50]
pKJ976	pCRPrtNeo with in-frame deletion of *mmp1283*	This study
pKJ1016	pCRPrtNeo with in-frame deletion of *mmp0600*	This study
pKJ1053	pCRPrtNeo with in-frame deletion of *mmp528*	This study
pKJ1056	pCRPrtNeo with in-frame deletion of *mmp0903*	This study
pKJ1068	pCRPrtNeo with in-frame deletion of *mmp0709*	This study
pKJ1104	pCRPrtNeo with in-frame deletion of *mmp0601*	This study
pWLG40	*hmv* promoter-*lacZ* fusion plus Pur cassette; Amp^r^	John Leigh
pKJ1007	pWLG40 with *mmp1283* complement	This study

### *M. maripaludis* Mutant Generation

The pCRPrtNeo plasmid derivatives carrying the inframe deletions of possible minor pilins were transformed into *M. maripaludis* Δ*flaK* using the PEG precipitation method [54]. Following growth overnight without selection, the transformation mixture was subcultured into McCas medium with neomycin to select for plasmid integration. Subsequently, the culture was used to inoculate McCas medium without antibiotic selection. After growth overnight in non-selective McCas medium, aliquots were plated onto 8-azahypoxanthine (240 µg/ml)-containing McCas-Noble agar plates and incubated at 37°C for a week in an anaerobic steel canister. Individual transformant colonies were subsequently picked and grown in Balch III medium. Cells from the various individual colonies were washed, resuspended in 2% NaCl and used as template for PCR using the respective sequencing primers (Table 1) which were designed to amplify across the targeted genes. Amplicons were analyzed by agarose gel electrophoresis to identify the smaller fragments expected of the deletion mutants. Potential mutants identified this way were restreaked for purity, single colonies again picked, grown in Balch medium III and screened by PCR with the sequencing primers.

### Complementation of the Deletion Strains

Each of the deleted genes was complemented by cloning the wildtype version of the gene under the control of the *nif* promoter in plasmid Complementation of the Δ*mmp1283* deletion strain.

Complementation of the Δ*mmp1283* gene deletion were done in pWLG40 [55] in which the complementing gene is under the control of the constitutive, strong *hmv* promoter [31], [56], [57]. For this, the *mmp1283* gene was amplified using the forward and reverse complementation primers (Table 1) containing added *Nsi*1 and *Mlu*1 restriction sites, respectively. The PCR product was digested with *Nsi*I and *Mlu*I and cloned into pWLG40, to generate pKJ1007. This plasmid was transformed into *M. maripaludis* Δ*mmp1283* using puromycin for selection.

### Electron Microscopy

Overnight cultures were washed with 50 mM MgSO_4,_ and negatively stained with 2% phosphotungstic acid. Cells were examined on Formvar-coated gold grids and imaged under a Hitachi 7000 electron microscope operating at an accelerating voltage of 75 kV.

## Results

**Figure 1:**
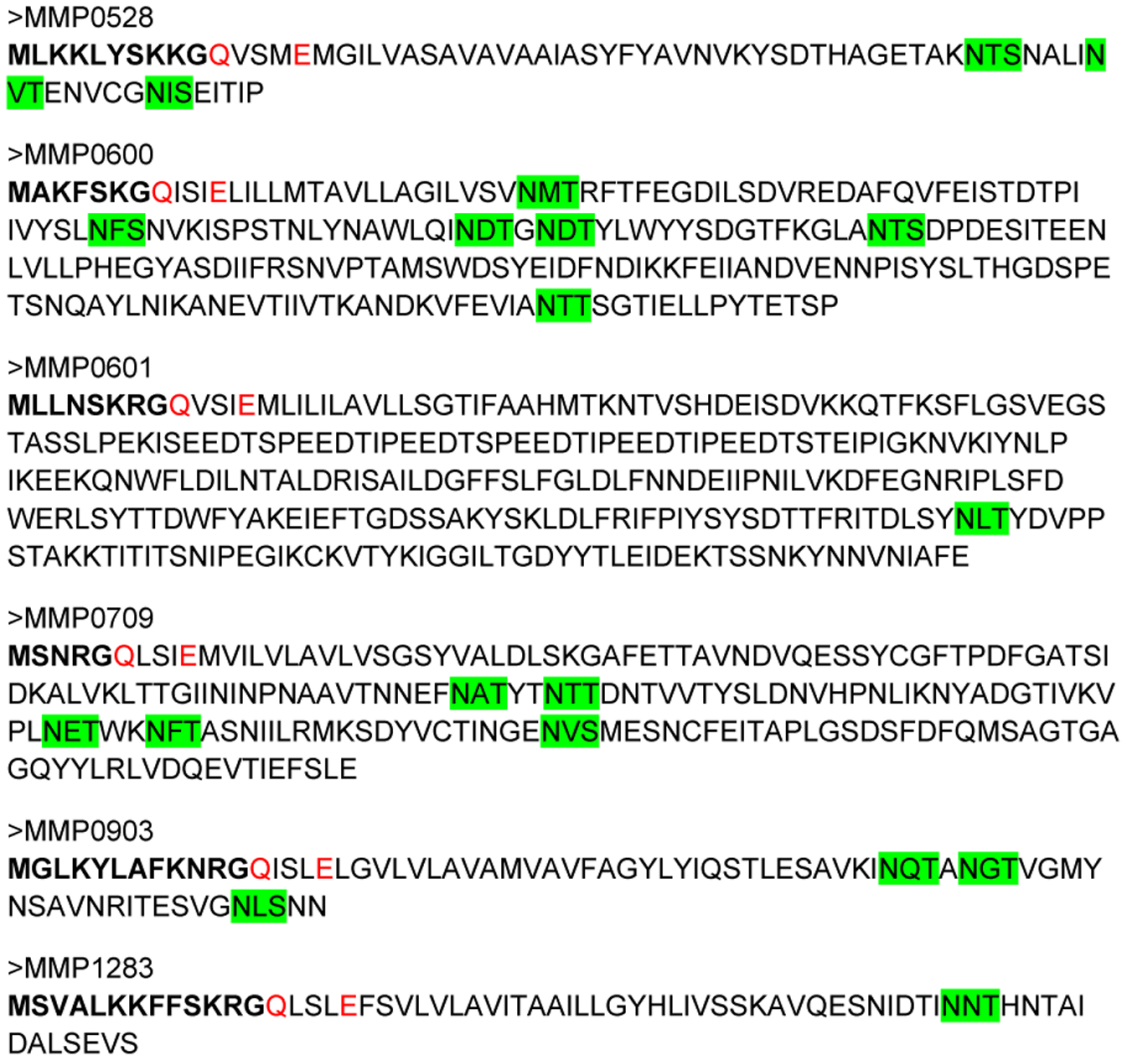
Sequences of the six pilin-like proteins of *M. maripaludis* studied in this report. The demonstrated or predicted signal peptides are shown in bold, conserved +1Q and +5E are shown in red and possible N-linked glycosylation sequons are highlighted in green.

Using the FlaFind program, Szabo et al. 2007 [32] identified 14 proteins in *M. maripaludis* that had class 3 signal peptides characteristic of archaellins and bacterial type IV pilins. Of these 14 genes, three were previously identified as archaellins [57] and several were already shown to be involved in pili formation [31]. The latter included *epdA, epdB* and *epdC* as well as the major pilin gene *mmp1685*. One of the 14 genes is a NAD+ synthase-related protein while the remaining six genes encode type IV pilin-like proteins with a DUF361 Pfam domain. The six pilin-like proteins were predicted to be processed by EppA, a prepilin peptidase that was already shown to process the DUF361 domain-containing pilins EpdA and EpdC [32]. The six genes under study here as encoding potential pili structural proteins are *mmp0528, mmp0600, mmp0601, mmp0709, mmp0903* and *mmp1283*. All of these proteins have a class 3 signal peptide of 5–13 amino acids ending with a glycine, a conserved +5 glutamic acid, and a conserved +1 glutamine (Figure 1), which may be required for EppA processing [32]. Of the six genes, *mmp0600* and *mmp0601* appear to be in an operon while the four remaining genes are not. MMP0528, MMP0903 and MMP1283 are very small proteins of 67–76 amino acids in length (54–63 amino acids after signal peptide removal), almost identical to the size of the major structural pilin, MMP1685 which is 74 amino acids in length (62 amino acids after signal peptide removal). The minor pilins already identified (EpdA, EpdB and EpdC) are about twice as long (130–156 amino acids). MMP0600, MMP0601 and MMP0703 are much larger proteins (200–299 amino acids). MMP1685 is known to be a glycoprotein with an attached N-linked pentasaccharide identical in structure to the tetrasaccharide identified attached to archaellins [58] but with an additional hexose attached as a branch to the linking sugar N-acetyl-galactosamine [31]. Analysis of the sequence of the 6 putative minor pilins identified 1–6 N-glycosylation sequons (N-X-S/T, where X is not proline) which indicates that all of these proteins could also be N-glycosylated (Figure 1).

**Figure 2:**
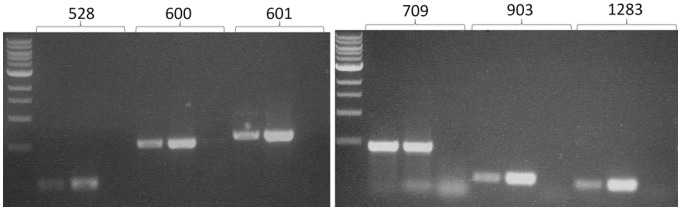
RT-PCR analysis reveals that all six pilin-like genes are transcribed under standard laboratory conditions. Primers were designed that would amplify an internal fragment for each of the six genes. For each gene, the triplet of lanes indicates PCR products obtained using as template either genomic DNA, purified RNA subjected first to reverse transcription or purified RNA not subjected to a reverse transcription step.

As a first step to determining that the 6 putative minor pilin genes corresponded to true genes, evidence for an mRNA transcript of each gene was sought using RT-PCR since detection of transcript provides support that an ORF indeed encodes a true protein [59]. RT-PCR was performed on isolated total RNA using primers that would amplify an internal fragment of each gene. In all cases, a PCR product was obtained of the predicted size only when the RNA was subjected to a reverse transcription step and not from the RNA sample itself (Figure 2), indicating that the product arose from cDNA and not contaminating genomic DNA present in the RNA preparation. The product of the RT-PCR was, in each case, identical in size to that obtained using genomic DNA as template. Sequencing of each PCR product confirmed their identities. Thus, all 6 putative pilin genes were transcribed under standard growth conditions in Balch medium III at 35°C.

**Figure 3:**
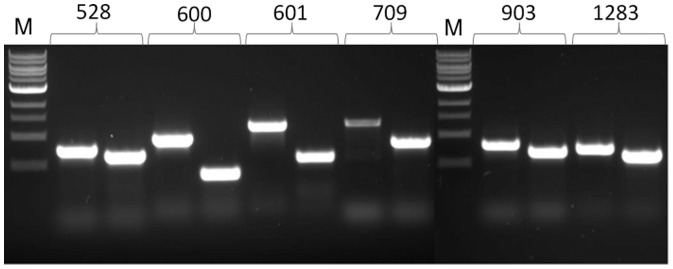
Confirmation of the deletion of each of the six pilin-like genes. PCR reactions used whole cells of the wildtype or the deletion strains as template with gene specific primers.In the case of each gene, the first lane is the PCR product obtained with wildtype cells as template and the second lane is the PCR product obtained with the deletion strain for that gene as template. In all cases a smaller PCR product is obtained for the deletion strain and the predicted sizes of the amplicons were obtained.

Each of the 6 genes was then targeted for deletion. The parent strain for these deletions was *M. maripaludis* Δ*flaK* [31]. This strain is deleted for *flaK* which encoded the prepilin peptidase essential for signal peptide removal from archaellins [60], [61]. Without this processing, the cells cannot assemble archaella so that the only surface structures remaining are pili. This makes analysis of effects on piliation by specific gene deletions easier to visualize. Transformants were screened using a PCR method with whole cells as template and primers that would amplify across the targeted genes. Successful deletion of each gene would result in a smaller PCR amplification product, whose size can be predicted from the site of the primers used in the PCR. Mutants carrying a deletion of each of the 6 putative minor pilin genes were obtained (Figure 3).

**Figure 4:**
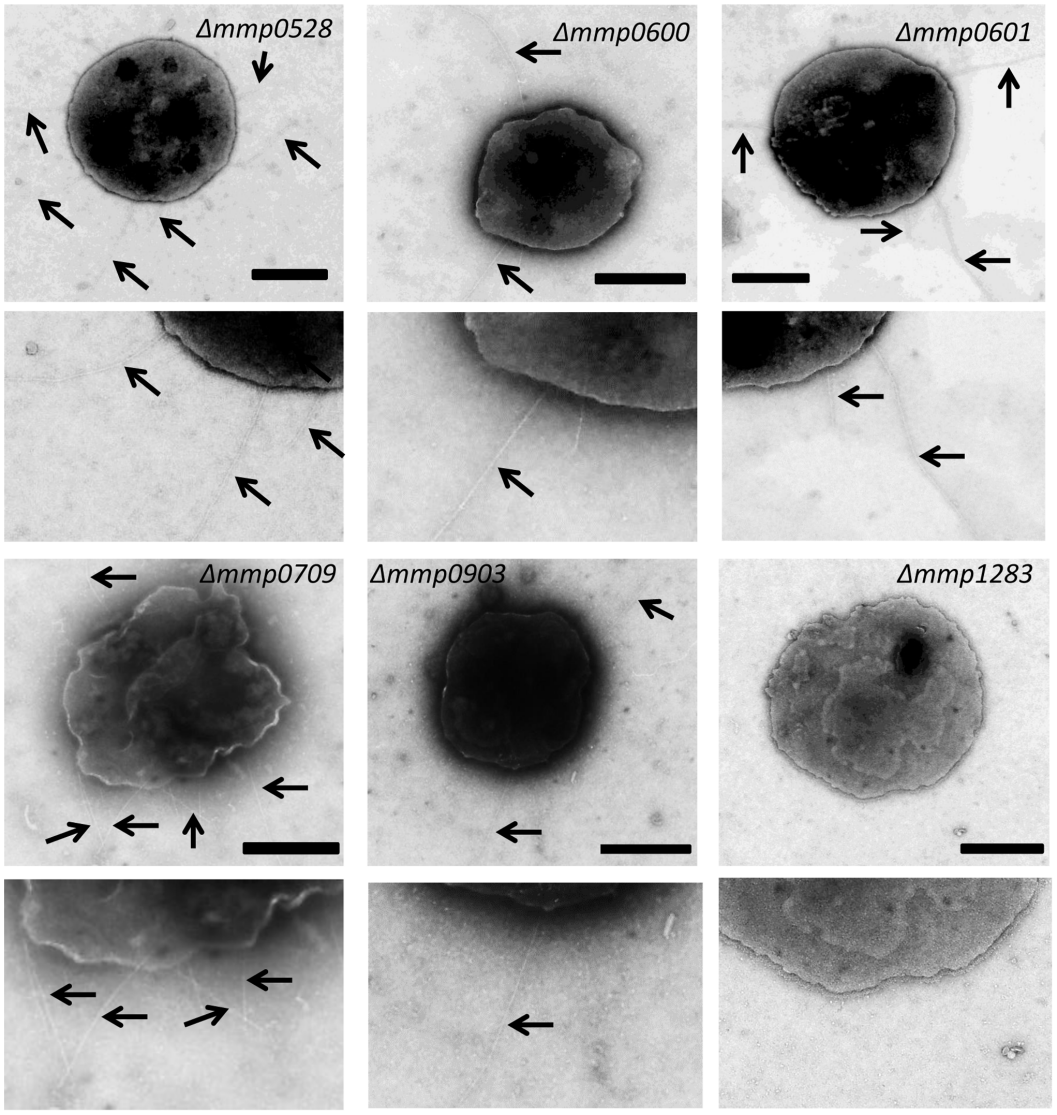
Electron micrographs of strains carrying deletions of each of the six pilin-like genes. An enlargement of a portion of each mutant cell is presented below the intact cell to enhance visualization of pili. Arrows indicate pili on the cell surface. Only the *M. maripaludis* Δ*mmp1283* strain is nonpiliated. Bar, 0.5 µm.

**Figure 5:**
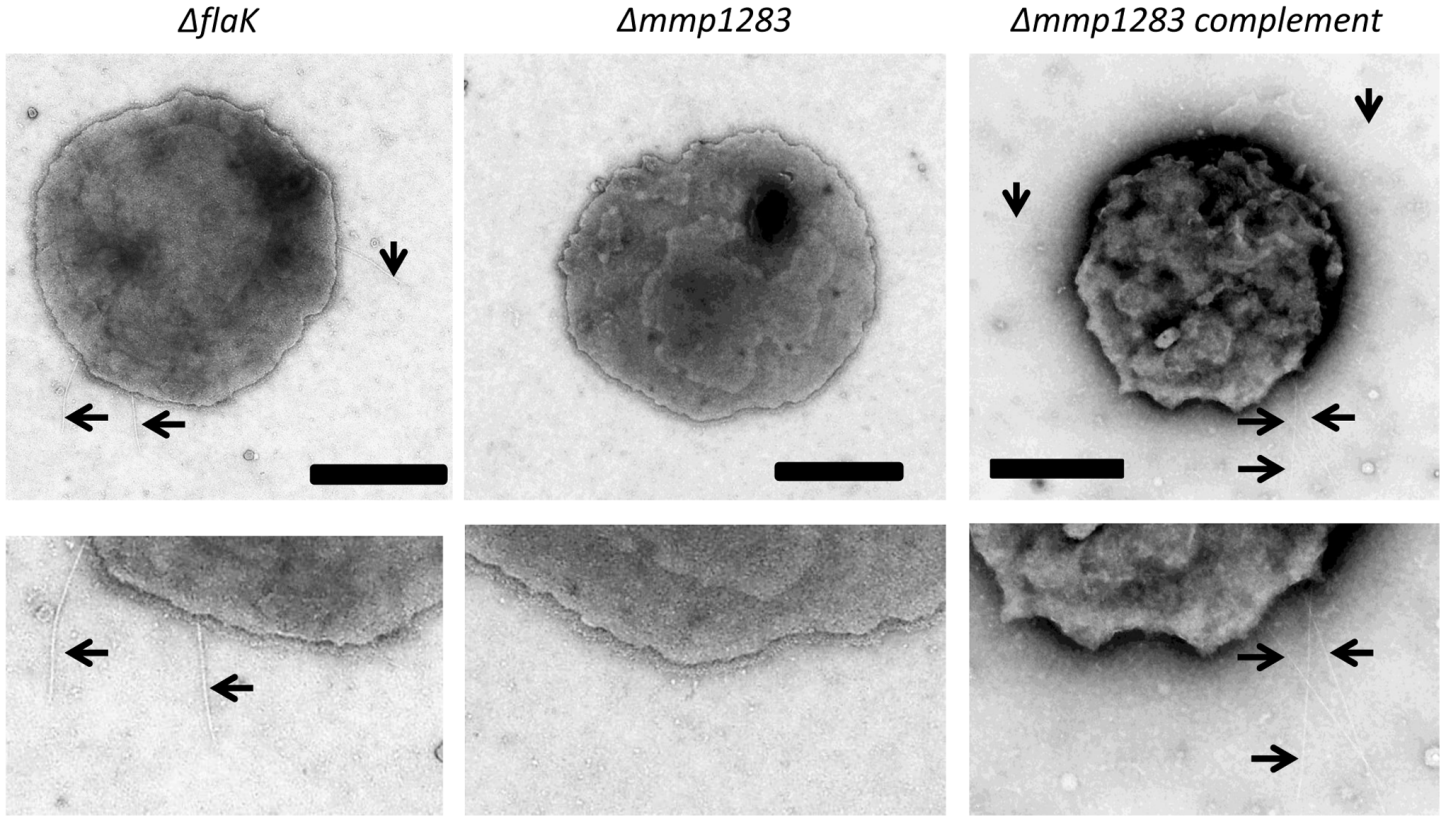
Electron micrographs showing that complementation restores piliation to the *M. maripaludis*Δ*mmp1283* strain. The *M. maripaludis* Δ*flaK* strain (non-archaellated) used as the parent for the pilin gene deletion studies is shown for comparison. An enlargement of a portion of each mutant cell is presented below the intact cell to enhance visualization of pili. The *M. maripaludis* Δ*mmp1283* strain was complemented with a plasmid-borne wildtype version of the *mmp1283* gene under the control of the constitutive *hmv* promoter. Bar, 0.5 µm.

To investigate whether any of the targeted genes played an essential role in piliation, all mutants were examined by electron microscopy for the presence and abundance of pili. In bacterial type IV pili systems usually there is one major pilin and a number of minor pilins [5], [20]. The major pilin, as well as three minor pilins, were already identified in *M. maripaludis* so it seemed unlikely that all six putative minor pilin genes studied here would be involved in assembly of the MMP1685 pili as this would result in a total of ten different structural proteins. The electron microscopic examination of the various mutants supported this contention as only the strain carrying the deletion of *mmp1283* was nonpiliated (Figure 4). Strains with deletions in *mmp0528, mmp0600, mmp0601, mmp0709* and *mmp0903* were all piliated to the extent of the parent *M. maripaludis* Δ*flaK* cells (compare piliation here to that seen in *M. maripaludis* Δ*flaK* cells in Figure 5). In the case of the *mmp1283* deletion strain, complementation with a wildtype version of *mmp1283* supplied in trans restored the cells to a piliation state comparable to *M. maripaludis* Δ*flaK* cells (Figure 5).

## Discussion

*Methanococcus maripaludis* is known to have at least two surface appendages that are assembled in a bacterial type IV pili mode, namely archaella and type IV-like pili [3], [26], [62]. Unusual for Archaea is that the processing of the structural subunits, i.e. archaellins and pilins, in *M. maripaludis* has been demonstrated to occur through the actions of two different prepilin peptidase-like enzymes whose substrate specificities do not overlap. FlaK processes only archaellins and EppA only pilins [32], [60], [63]. In other studied Archaea, a single enzyme designated PibD is thought to remove the signal peptide from all pilin-like substrates, including archaellins [25], [64], [65]. As more Archaea are studied, it seems likely that the division of labor in processing prepilin-like substrates by two separate prepilin peptidase-like enzymes reported so far only in *M. maripaludis* may be found in other members of the domain. It has been reported that several members of the Euryarchaeota, mainly Methanococcales as well as *Pyrococcus* and *Thermococcus* species harbor both *flaK* and *eppA* homologs in their genomes [32]. There are also a limited number of Euryarchaeota that have been reported to possess more than one copy of *flaK* [66] but the roles and substrates of these potential prepilin peptidases, some found in species reported to be non-archaellated cells, has not been studied.

Previous studies have demonstrated roles for four pilins in the biosynthesis of the *M. maripaludis* pili. Genes encoding three minor pilins, EpdA, EpdB and EpdC are found in a single large gene cluster [32] that also includes EppA and several other genes shown to be essential for pili formation (Nair et al., submitted). Deletions of the genes for the three pilins result in either completely nonpiliated cells or cells in which the number of pili is significantly reduced [31]. The major structural protein was identified as MMP1685 and deletion of *mmp1685* led to nonpiliated cells. Interestingly, MMP1685 had been previously identified by bioinformatics analysis as a predicted substrate for EppA [32]. In addition, that study also predicted that six other genes encoded pilin-like proteins likely to be EppA substrates. In this report, deletions were created in all six genes to investigate the potential role of the encoded pilin-like proteins in the biosynthesis of the surface pili of *M. maripaludis*.

Three of the pilin-like proteins MMP0528, MMP0903 and MMP1283 are of similar size to the previously identified major pilin structural protein MMP1685 [31] while the other three (MMP0600, MMP0601 and MMP0709) are much larger. The smaller pilin sizes are typical lengths for type IV pilins of the Flp (Tad) class and the presence of +1 glutamine is also common in Gram positive Flp pilins [1], [5], [6]. *M. maripaludis* pilins, however, lack the +6 tyrosine and so called Flp motif of Flp pilins. All six pilin-like proteins possess a +5 glutamic acid, conserved in most bacterial type IV pilins [5] but absent in the pilins of both *Sulfolobus* [2], [30] and *Haloferax* [48]. Examination of mutant strains carrying deletions of each of the targeted genes by electron microscopy indicated that only *mmp1283* was essential for piliation as all other deletion strains had similar numbers of pili per cell as wildtype cells. A piliated state could be restored to the Δ*mmp1283* strain by supplying a wildtype copy of the gene in trans under the control of a constitutive *hmv* promoter. Like the major pilin MMP1685 and all the previously identified minor pilins (EpdA, EpdB, and EpdC), MMP1283 carries an amino acid sequon necessary for N-linked glycan attachment, suggesting that MMP1283 may be modified by the pentasaccharide found attached to MMP1685 [31]. While the *M. maripaludis* major pilins are modified with the N-linked glycan, this posttranslational modification is not needed for pilus formation as pili are formed even in an *aglB* mutant that is missing the oligosaccharyltransferase necessary to transfer the glycan from its lipid carrier to the protein target [67]. It is not yet known if these assembled pili, however, are functional in surface attachment, the only known function attributed to the pili [62].

The function of the other pilin-like genes shown not to be essential for the MMP1685 pili is unknown but several possibilities exist. They could still be involved in the MMP1685 pili structure but dispensable proteins. In *Neisseria meningitidis* type IV pili, five different pilin proteins are necessary for piliation while three others that are normally incorporated into the pilus are dispensable for pili formation but play important roles in function. Interestingly, these three minor pilins (PilX, ComP and PilV) all have different functions [68]. Alternatively, the pilin-like proteins could be structural proteins of an entirely separate pilus-like structure. It is known in the thermoacidophilic archaeon *Sulfolobus acidocaldarius* that two different types of type IV pili are made [25], [30], [47] with one type (Ups pili) only made after UV induction or other DNA damaging treatment [29], [47]. Perhaps, under still undefined growth conditions or stress, *M. maripaludis* has the capacity to assemble a novel pilus type composed of one or more of the remaining pilin-like proteins that currently have no function. Although mRNA transcripts were detected for all five of the other pilin-like genes by RT-PCR, it is possible that posttranscriptional regulation mechanisms prevent pilin protein synthesis. The regulation of archaella assembly, for example, in *S. acidocaldarius* seems to involve both transcriptional and posttranscriptional control [25], [69]. Alternatively, in the case of the five pilin-like proteins for which deletion did not affect formation of MMP1685 pili, other key components essential for pili formation from these pilin-like proteins may not be made under tested growth conditions, even though the pilins themselves are made. In the case of *S. acidocaldarius,* archaella synthesis is induced under tryptone starvation conditions even though archaella core proteins are constitutively produced. This is because the major structural protein (the archaellin FlaB) is only made under starvation conditions [25]. A third possibility is that *M. maripaludis* makes a very short pilus-like structure from one or more of these proteins that has gone undetected by electron microscopy. In *Sulfolobus solfataricus*, it is known that sugar binding proteins are pilin-like glycoproteins that form a macromolecular cell-surface-associated structure [25], [33] that may form a short pilus-like structure that extends only from the cytoplasmic membrane to the S-layer [3]. The putative bindosome pilus-like structure has never been observed in electron microscopic studies. Type two secretion systems in bacteria use type IV pilin-like proteins to produce a very short pilus-like piston (pseudopilus) proposed to push exoproteins through an outer membrane channel [70]. This pseudopilus would extend only from the cytoplasmic membrane to the outer membrane and likely be dynamic in nature. While *M. maripaludis* does not utilize sugars as substrates or possess a type II secretion system, there may be other functions in the cells that may require such a short type IV pilus-like structure. The recent identification of putative diverse type IV pili in a variety of Gram positive bacteria using a program called PilFind [1] suggest that all possible functions for these structures have not likely been identified yet.

In bacteria, it is not unusual for type IV pili to be composed of a major pilin and multiple minor pilins. For example, in *P. aeruginosa*, the pilus is comprised of the major pilin PilA and five minor pilins (FimU, PilV, PilW, PIlX and PilE) which were all shown to be incorporated into the pilus by immunogold labelling experiments [18]. Among the studied Archaea, however, the type IV pilus locus of *M. maripaludis* appears to be considerably more complex than the two gene clusters encoding Aap and Ups pili in *Sulfolobus*. In both the Aap and Ups pili systems, there appear to be only two pilin genes and they are encoded along with the conserved ATPase and membrane component genes. Interestingly, in the case of Aap pilins of *S. acidocaldarius* and Ups pili of *S. solfataricus*, the lengths (138–168 amino acids with signal peptides) are much larger than seen with MMP1685. In the case of the six recently described *Hfx. volcanii* pilins, none are co-transcribed with the pilus ATPase and conserved membrane protein genes [48]. The *M. maripaludis* pili genes are known to lie now in at least four separate locales around the chromosome. One major operon encoding EppA, EpdA, EpdB and EpdC along with other essential genes has already been analyzed to some extent [31], [32]. Furthermore, there is a separate locus encoding the ATPase and two membrane protein components (Nair et al., submitted) as well as the major pilin subunit MMP1685 [31] and now, in this report, the minor pilin MMP1283. It is not yet known if type IV pili formation is constitutive in *M. maripaludis* or whether it can be induced or repressed under specific environmental circumstances such as attachment or planktonic conditions, as is observed in bacterial type IV pili systems [5]. If pili formation is not constitutive then the cells must regulate transcription of several essential gene clusters located at some distance from each other. Regulation of minor pilin expression has not been well studied even in bacteria. In type IVb systems where minor pilins are clustered with other components of the pilus system, they are likely co-regulated with them. However, in type IVa systems, minor pilins are often unlinked to other pilus component genes, as found in *M. maripaludis*, and they can be differentially regulated, sometimes by two-component systems [5], [6], [71].

In *Sulfolobus* species, studies on the regulation of pili have already been initiated, with intriguing findings reported. In *S. acidocaldarius,* a two component regulatory system (ArnA and ArnB) was found to repress archaella expression. Interestingly, overproduction of ArnA also resulted in a strong enhancement of Aap pili production, suggesting there is a regulation of the two surface organelles that involves cross-talk between the two systems [69]. Recently, the product of an Lrs14 regulator gene *saci0446*, was shown to bind to promoters of both archaellum (*fla*) genes and *aap* pili genes and result in an upregulation of *aap* genes and downregulation of *fla* genes, again showing that regulation of different surface structures in this archaeon is connected [46].

This report adds to our knowledge about the complexity of the type IV-like pili in *M. maripaludis* by identifying a fourth minor pilin that is essential for piliation. Unlike other archaeal systems, piliation in *M. maripaludis* requires a separate pilin-specific signal peptidase, two conserved membrane (platform) proteins), four minor pilins and additional novel essential proteins [31], [32]. This report also eliminates five other pilin-like proteins as playing an essential role in MMP1685 pili formation and hints that they may form additional type IV pili-like surface structures under appropriate growth conditions.
